# A systematic review on the psychological factors behind vaccine hesitancy in the COVID-19 era

**DOI:** 10.3389/fpubh.2025.1711428

**Published:** 2025-11-11

**Authors:** Francesco Panico, Rosalia De Biase, Laura Catalano, Isa Zappullo, Francesca D'Olimpio, Luigi Trojano, Laura Sagliano

**Affiliations:** Department of Psychology, University of Campania “Luigi Vanvitelli”, Caserta, Italy

**Keywords:** vaccine hesitancy, emotion, cognition, personality, COVID-19, health, intervention

## Abstract

**Background:**

Vaccine hesitancy may represent a global threat because of its inherent consequences for health, social and economic systems. Understanding the factors associated with vaccine hesitancy is fundamental to developing effective healthcare policies. While previous studies have mainly focused on sociological and cultural variables and transient illness-specific fears and beliefs, the present systematic review focuses on the psychological factors (such as emotional dispositions, cognitive functioning and expectations, and stable personality traits) associated with vaccine hesitancy during the COVID-19 era.

**Methods:**

A systematic review using a systematic search of PubMed, PsychINFO and Web of Science databases was performed with a time frame ranging between 1 January 2020 to 31 January 2025 focusing on psychological factors and vaccine hesitancy. Studies targeting the general population and employing validated instruments to assess emotional, cognitive and personality factors and vaccine hesitancy were selected, while investigations on context-specific, psycho-social, cultural and political factors were excluded. Quality and risk of bias in the selected studies was assessed using an adapted version of the Newcastle-Ottawa Scale, and main studies’ characteristics, variables and outcomes were synthesised using a narrative approach and table.

**Results:**

Fourteen studies were finally included in the qualitative synthesis. The results showed that some variables such as depressive and anxiety levels, as well as emotion regulation strategies may affect vaccination behaviour, although some cultural and generational differences were also observed. Differences in cognitive flexibility, decision-making, and personal expectations may influence vaccine hesitancy. Notably, some personality factors, like extraversion, openness, conscientiousness and dark personality traits, may influence hesitancy to vaccinate.

**Conclusion:**

This review highlights emotional, cognitive, and personality factors associated with vaccine hesitancy, providing evidence for personalised, evidence-based interventions aimed at promoting adherence to national vaccination policies.

## Introduction

Vaccine hesitancy, defined as a delay in acceptance or refusal of vaccination despite availability of vaccination services, has been identified by the World Health Organisation (WHO) as a significant global health threat (1, 2). This multifaceted phenomenon is influenced by individual characteristics, historical context, and geographical location (3–5).

The widespread recent pandemic waves of COVID-19 dramatically brought the issue of vaccine hesitancy to the forefront of public health discourse and policy, for its severe consequences on the social, economic, and cultural landscapes and for the inherent impact on the global health (3, 4). In this context, vaccination has been recognised as a fundamental strategy against the detrimental consequences of the pandemic. However, an ample segment of the population expressed reluctance towards or opposition to vaccination, posing significant challenges to achieving widespread immunity and limiting the health system ability to contain the virus spread (6). A rapid diffusion of both information and misinformation, defined as “infodemic,” made the decision-making policies about vaccination increasingly complex and arduous (7, 8).

Previous research identified several factors associated with vaccine hesitancy. Common frameworks, such as the 3C model (9), identify three possible determinants: confidence (i.e., trust in vaccine safety, efficacy, and delivery systems), complacency (i.e., low perceived risk of disease), and convenience (i.e., accessibility of services). Trust in science, medical institutions, and governmental authorities, along with an individual’s prior vaccination history and perceived severity of the disease, are thought to be key factors in reducing vaccine hesitancy (1, 3, 10). Frequently mentioned reasons for reluctance are concerns regarding vaccine safety, potential side effects, and doubts about vaccine efficacy and necessity. Moreover, factors such as “chance externality” in health locus of control (namely a belief that one’s health depends on fate), anxiety symptoms, perceived psychological status, and even lower self-esteem and high perceived stress have been shown to influence vaccination intentions (1, 11). Additional factors include the influence of significant others, conspiratorial beliefs, and reliance on social media for information (7, 12).

Several systematic reviews have previously synthesised the psychological and social aspects contributing to vaccine hesitancy in the context of COVID-19. For instance, Pourrazavi et al. (13) showed that the most common reason for vaccine hesitancy were lack of confidence and complacency towards vaccinations, together with multiple other factors including concerns about vaccine safety and side effects, perceived susceptibility and severity to illness, and social and peer influence, which could influence delay or refusal to accept the vaccine. Romate et al. (10) identified factors such as the appraisals of the COVID-19 pandemic, vaccine safety and side effects, vaccine confidence/trust, misinformation and mistrust in government and healthcare professionals, as major issues contributing to vaccine hesitancy. Similar results were reported by Rizzo et al. (4) when reviewing the psycho-social dimensions of vaccine hesitancy in Europe and the United States after vaccine availability, and by Blukacz et al. (5) who focused on determinants of vaccine confidence in low- and middle-income countries using qualitative evidence.

However, the syntheses described above largely focused on understanding the immediate beliefs, perceptions, and situational factors contributing to vaccine hesitancy, or on the socio-cultural aspects associated with vaccination behaviour (4, 5, 10, 13). A comprehensive understanding of the phenomenon should also delve into individuals’ stable and intrinsic psychological characteristics. Thus, in the present work, to the aim of complementing previous research on this issue, the term ‘psychological factors’ is used to refer to enduring *psychological* dimensions rather than to denote other *psycho-social* determinants. Indeed, enduring psychological dispositions are not specific to a particular health or vaccination context but manifest across multiple aspects of everyday life (14). These individual-centred, rather than disease/vaccination-centred, psychological factors are known to influence several health behaviours (15–18), which might include the decision to vaccinate. Investigating these deeper and stable psychological determinants is crucial for public safety and health, as it allows for the development of more targeted and effective interventions and for strengthening responses to face pandemic threats.

The present systematic review addresses this gap by synthesising the most recent literature on the role of stable, intrinsic psychological characteristics in influencing vaccine hesitancy, not restricted to vaccination-specific emotional responses, attitudes or socio-cultural variables. By this approach the present paper aims at offering a more nuanced and comprehensive understanding, crucial for adjusting public health strategies.

## Methods

The systematic review conformed to the guidelines described in the Preferred Reporting Items for Systematic Reviews and Meta-Analyses (PRISMA) initiative^1^ for bibliographic research and data communication in systematic reviews (19).

### Search strategy

For selecting the relevant studies, a systematic search was performed on the databases PubMed, PsychINFO and Web of Science. The search strategy targeted studies with a time frame ranging 1/1/2020–31/1/2025. The timeframe selected for this study was determined to retrieve the most recent literature on the subject, considered the sizable surge in interest regarding the factors associated with vaccine hesitancy during and after the period of the COVID-19 pandemic. However, the search was not strictly related to COVID-19 vaccination, but potentially embraced vaccine hesitancy towards other medical conditions. The following search string was used and adapted to the specific databases: “(vaccination OR vaccines) AND (refusal OR opposition OR hesitancy OR reluctance OR rejection OR non-adherence OR “non-adherence” OR resistance OR scepticism) AND (“psychological antecedents” OR “psychological antecedent” OR “psychological predictors” OR “psychological predictor” OR personality OR obsess* OR paranoi* OR phobi* OR emotion* OR anxiety OR “health anxiety” OR fear OR mood OR worry OR belief* OR “locus of control” OR self-efficacy OR “self-efficacy” OR stress OR self-regulation OR “self-regulation” OR moral* OR responsibility OR optimism OR “risk perception”).”

### Eligibility criteria

Studies were considered eligible if they: (i) assessed vaccination hesitancy or acceptance in humans, (ii) included participant samples covering the general population, (iii) adopted standardised and validated instruments to assess psychological and common psychopathological symptoms and vaccine hesitancy, (iv) were published in English language in peer-reviewed journals and reported original empirical data.

Exclusion criteria were: (i) studies involving specific group of individuals including parents, healthcare professionals, specific ethnic groups and minorities or clinical populations with peculiar neurological, psychiatric or physical disorders, (ii) studies investigating state-like emotional reactions or context-specific attitudes (e.g., fear of COVID-19, anxiety about side effects of drugs), (iii) studies focused on social, cultural, political, or economic factors associated with vaccine hesitancy, (iv) narrative reviews without original data, conference abstracts, unpublished reports, and doctoral theses.

The restriction of the study search to the general population, and the exclusion of specific professional, social, and clinical groups, were intended to ensure that the factors identified reflected enduring and general psychological characteristics rather than context-dependent attitudes or motivations linked to particular social, occupational or clinical circumstances.

### Studies selection process

**Figure 1:**
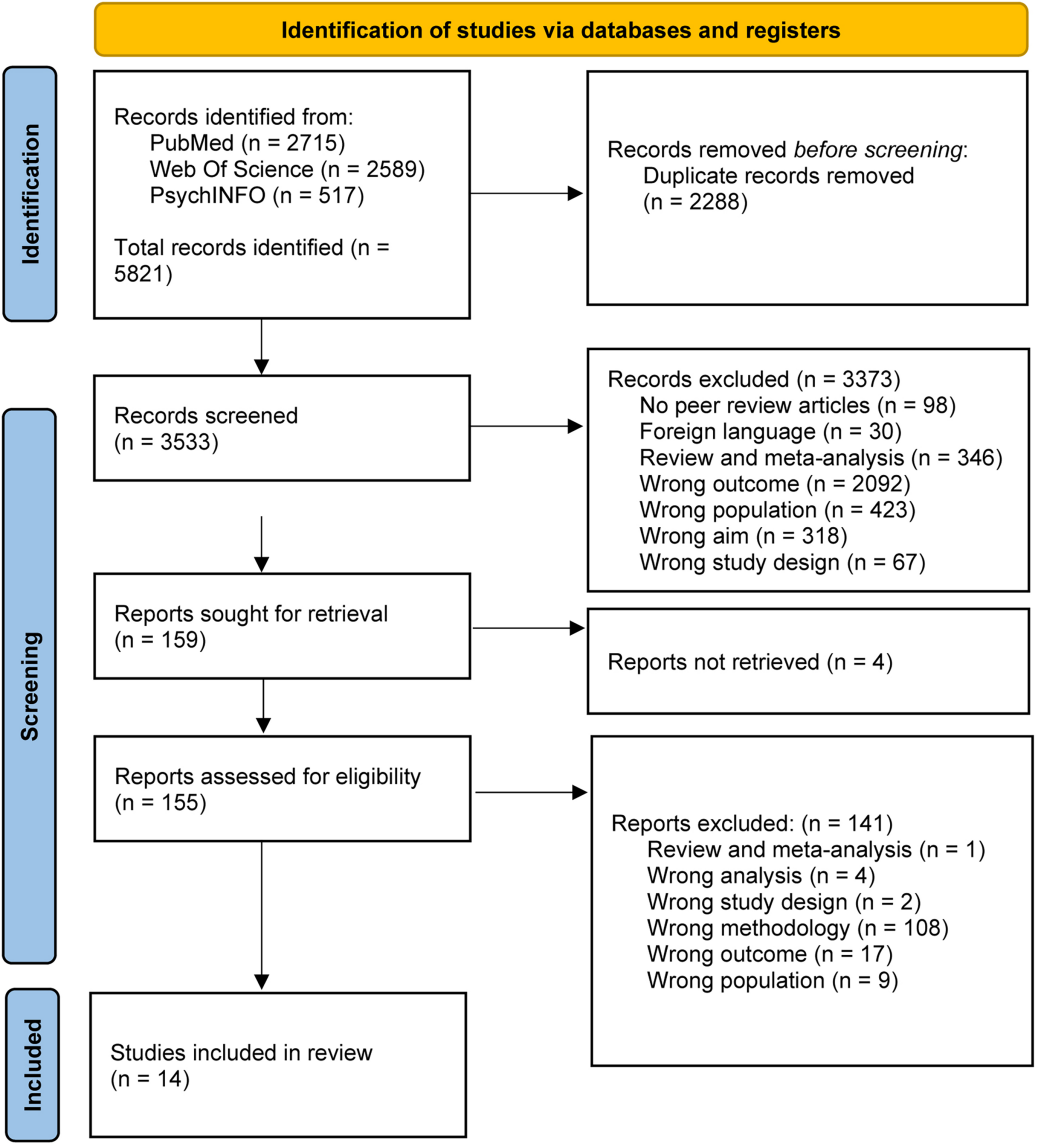
Flowchart illustrating the identification of studies via databases and registers.

The process of study selection is described in the PRISMA diagram (Figure 1). Retrieved studied were imported and screened for duplicates by automated tool (Rayyan—https://www.rayyan.ai—a web and mobile app for systematic review) (20). Then articles were screened by title and abstract for relevance to the study variables. Finally, full text studies were analysed, and relevant information extracted (see below). Included studies were then rated for quality of evidence. The selection and screening process, quality assessment as well as data extraction were performed by two independent reviewers (RDB, LC) and a third reviewer (IZ) intervened in case of disagreement.

### Data extraction and synthesis

The results of the included studies were synthesised using a narrative descriptive approach. Data were systematically extracted and organised into a summary table reporting key study characteristics (authors and year of publication, sample characteristics, study design, methods used for assessing vaccine hesitancy and psychological variables, and main results; Table 1). The rationale for adopting this approach was to ensure methodological transparency and to integrate diverse forms of evidence in a coherent and comparable framework (21), while the heterogeneity in study designs, psychological constructs assessed, and outcome measures, made a quantitative meta-analysis not appropriate.

**Table 1:** Studies description for each psychological domain identified (emotion, cognition and personality).

Study	Design	Sample (numerosity, sex, age, education, and nationality)	Vaccine hesitancy measure (1)	Psychological measures (2)	Psychological construct	Main results	NOS-quality assessment
Emotional dispositions, coping strategies, and specific fears							
Freeman et al. (28)	Correlational	*N* = 15,014 (51.3% F, age range = 18–99 years, education = from no qualification to post graduate education) United Kingdom	OCVHS	SPS, MFS	Blood injection injury fear	Blood injection injury fear was associated with vaccine hesitancy towards COVID-19	7 * - good
Harada and Watanabe (40)	Correlational	*N* = 1,000 (86.7% F, age range = 20–70 + years, education = postgraduates and undergraduates) Japan	VHS	Three-factor Anxiety Scale	General anxiety	General anxiety did not predict attitude towards COVID-19 vaccination. General anxiety associated with vaccine acceptance	5 * - satisfactory
Jayakumar et al. (39)	Correlational	India: *N* = 372 (63.7% F, age range = 18–53 years, education = postgraduates and undergraduates); Saudi Arabia: *N* = 305 (65.6% F, age range = 18–58 years; education = postgraduates and undergraduates) India and Saudi Arabia	VHS	PHQ-2, GAD-2, IES-6	Depression, anxiety and PTSD	In Saudi Arabia: higher hesitancy towards COVID-19 vaccines predicted increased depression, anxiety, and perceived need for mental health support, and vice versa. In contrast, in India, vaccine hesitancy was only associated with increased anxiety, while no mental health variables significantly predicted hesitancy	5 * - satisfactory
Marschalko et al. (31)	Correlational	*N* = 978 (100% F, Gen Z: Mage = 21.31 SD = 1.85, Gen Y: Mage = 34.92, SD = 4.88, Gen X: Mage = 49.62, SD = 5.21, education = from grades to doctoral degree) Romania	CoVaH	PPFI	Personal psychological flexibility	Psychological flexibility showed a limited but relevant role in predicting COVID-19 vaccine hesitancy, with significant effects emerging only in women from GenY. Specifically, avoidance was a significant negative predictor of vaccine uptake, indicating that lower psychological flexibility is associated with greater vaccine hesitancy in this age group. In contrast, none of the psychological flexibility components (avoidance, acceptance, harnessing) were significantly associated with vaccination behaviour in women from Gen Z or Gen X (≥42 years)	5 * - satisfactory
Mcneil and Purdon (24)	Cross-sectional and correlational	*N* = 148 (52 in the control group, 77.1% F; Mage = 30.72, SD = 12.24, education not reported) Canada	VHS	HPRS, IUS-SF, Disgust propensity and sensitivity scale	Reactance to restrictions on freedom of choice, intolerance of uncertainty, disgust propensity and sensitivity scale	In the control non anxious group, higher intolerance of uncertainty predicted greater COVID-19 vaccine hesitancy. (In the anxious group, intolerance of uncertainty was slightly negatively associated with vaccine hesitancy)	4 * - unsatisfactory
Omar et al. (30)	Correlational	*N* = 360 (50.6% F, age range = 19–65 years; education = matric, intermediate, graduation, post-graduation) Pakistan	OCVHS; VAI	HADS	Levels of depression and anxiety	Anxiety, and depression were strongly associated with both COVID-19 vaccine hesitancy and vaccine acceptance in opposite directions. Correlational analyses showed that vaccine hesitancy was significantly and negatively associated with anxiety, and depression, while vaccine acceptance was positively associated with the same variables. Depression contributed significantly to acceptance, while anxiety was not a significant predictor	4 * - unsatisfactory
Veronese et al. (32)	Correlational	*N* = 1,122 (772 F, Mage = 40.83, SD = 8.8, education = bachelor’s degree, master’s degree and up to secondary school) Palestine	VAC-COVID-19	DASS-21	Depression, stress and anxiety	COVID-19 vaccination reluctance is positively predicted by symptoms of depression in the West Bank; in the East Jerusalem and Israeli Palestinians symptoms of stress positively predicted the vaccine’s reluctance	5 * - satisfactory
Vicario et al. (23)	Cross-sectional study with between-group comparisons	*N* = 120 (70 F, age range = 18–38 years, education = university master students) Italy	aVHS	STAI-Y1, Y2, TAS-20, DPSS-R	Alexithymia, state and trait anxiety, disgust sensitivity and propensity	No significant predictive value was found for anxiety, alexithymia, or disgust sensitivity	4 * - unsatisfactory
Cognitive factors, expectancies, and beliefs							
Demirci et al. (25)	Quasi-experimental	Vaccinated (*N* = 70, 60% F, Mage = 28.99, SD = 9.25, Medu = 14.46, SD = 3.18); Non-Vaccinated (*N* = 70, 53.3% F, Mage = 31.94, SD = 10.05, Medu = 13.51, SD = 3.68) Turkey	SVH	IGT, BAI, BDI, BIS-11-SF	Decision making ability, anxiety, depression and impulsivity levels	Non-vaccinated participants made more choices from risky decks during IGT-5 block. Statistically significant negative correlation between IGT-5 block and vaccine hesitancy towards COVID-19	4 * - unsatisfactory
Gialama et al. (27)	Correlational	*N* = 300 (190 F, age range = 20–84, education = tertiary, secondary, primary education) Greece	aVHS	LOT-R	Optimistic or pessimistic expectations	Dispositional optimism was a significant negative predictor of the “lack of confidence” component of vaccine hesitancy: lower optimism scores were associated with higher distrust in vaccines	5 * - satisfactory
Gomes-Ng et al. (26)	Correlational	*N* = 601 (343 F, Mage = 32.9, SD = 11.58, education = high school, tertiary certificate or diploma, bachelor’s degree, postgraduate degree) New Zealand	MVHS	WCST	Cognitive inflexibility	Lower cognitive flexibility (more perseverative errors) predicted greater personal vaccine hesitancy, but not external hesitancy	4 * - unsatisfactory
Pellegrini et al. (29)	Correlational	*N* = 252 (70% F, age range = 18–67, education = secondary, undergraduate, postgraduate, doctorate) United Kingdom	OCVHS	WCST, OCI-R, CPAS, DASS-21	Cognitive inflexibility, obsessive-compulsive traits, obsessive personality, depression, anxiety, stress and general distress	Cognitive inflexibility, measured through perseverative errors on the WCST, significantly predicted COVID-19 vaccine hesitancy. None of the self-reported psychological measures were associated with hesitancy, suggesting that only objectively assessed cognitive inflexibility was a significant predictor	4 * - unsatisfactory
Personality factors, interpersonal reactiveness and individual differences in interoceptive awareness							
Giancola et al. (36)	Correlational	*N* = 210 (50% F, age range = 18–71, Medu = 14.21, SD = 2.67) Italy	VAES	DTDD	Psychoticism, narcissism, machiavellism	Psychoticism, narcissism and machiavellism were positively correlated with COVID-19 vaccine hesitancy. Although this dark triad did not predict vaccine hesitancy directly, conspiracy beliefs and COVID-19 risk perception sequentially mediated the association between the dark triad and hesitancy	5 * - satisfactory
Panish et al. (12)	Correlational	Not retrieved United States	VH	Big Five Inventory 2-XS	Openness, agreeableness, conscientiousness and negative emotionality	Openness and agreeableness covary with less COVID-19 vaccine hesitancy. Conscientiousness shows a similar pattern of relationships. Extraversion correlates with more vaccine hesitancy. Negative emotionality is largely unrelated to vaccine hesitancy	5 * - satisfactory
Vicario et al. (23)	Cross-sectional study with between-group comparisons	*N* = 120 (70 F, age range = 18–38 years, education = university master students) Italy	aVHS	MAIA-2, IRI	Interoception, empathy	Individuals with lower cognitive empathy (IRI) and lower score on the not distracting sale of MAIA showed significantly greater vaccine hesitancy	4 * - unsatisfactory

(1) SVH, Scale of Vaccine Hesitancy; OCVHS, Oxford COVID-19 Vaccine Hesitancy Scale; aVHS, Adult Vaccine Hesitancy Scale; VAES, Vaccination Attitudes Examination Scale; MVHS, Multidimensional Vaccine Hesitancy Scale; VHS, Vaccine Hesitancy Scale; CoVaH, Multidimensional Covid-19 Vaccine Hesitancy Scale; VAI, Vaccine Acceptance Instrument; VH, Vaccine Hesitancy; VAC-COVID-19, COVID-19 Vaccine Acceptance Scale. (2) IGT, Iowa Gambling Task; BAI, Beck Anxiety Inventory; BDI, Beck Depression Inventory; BIS-11-SF, Barratt Impulsivity Scale Short Form; SPS, Specific Phobia Scale, MFS, Medical Fear Survey; LOT-R, Revised Life Orientation Test; DTDD, Dark Triad Dirty Dozen; WCST, Wisconsin Card Sorting Task; PHQ-2, Patient health questionnaire-2, GAD-2, Generalised Anxiety Disorder-2, IES-6, Impact of Event Scale-6; PPFI, Covid-19 Health-related Personal Psychological Flexibility Index; HPRS, Hong’s Psychological Reactance Scale; IUS-SF, The Intolerance of Uncertainty Scale Short Form; HADS, Hospital Anxiety and Depression scale; OCI-R, Obsessive- Compulsive Inventory-Revised, CPAS, Compulsive Personality Assessment Scale; DASS-21, Depression Anxiety Stress Scale-21; STAI, State–Trait Anxiety Inventory; TAS-20, Toronto Alexithymia Scale 20; MAIA-2, Multidimensional Assessment of Interoceptive Awareness; IRI, Interpersonal Reactivity Index; DPSS-R, Disgust Propensity and Sensitivity Scale-Revised.

### Quality assessment

The Newcastle-Ottawa Scale (NOS) (22) adapted for cross-sectional study was used to assess quality of evidence. The scale is aimed at evaluating appropriateness of sample selection, outcome, and statistical comparisons processes in cohort and single cases studies. Based on a star scoring system, the NOS classifies studies as “very good” (9–10 stars), “good” (7–8 stars), “satisfactory” (5–6 stars) and “unsatisfactory” (0–4 stars). In the version of the scale used in this study (see Supplementary materials), the score system and some items were adapted for their use in cross-sectional studies. Specifically, the original items were modified by incorporating an evaluation of the sample size calculation, of the psychological measures used in the study, of the blinding procedure adopted, of the possible confounding factors on data, and of appropriateness of the statistical approach. This version of the scale was selected because most of the included studies were cross-sectional, but the same adapted version of the NOS was also applied for quasi-experimental design or between-group comparisons as the scale integrated relevant domains common across psychological research (selection of the sample, measurement of variables and outcomes, confounding factors and statistical analyses).

For each study included in the review, two independent researchers (RDB and LC) performed quality assessment. Any discrepancy was solved by a third reviewer (IZ).

## Results

Among the 5,821 articles from the primary search, 2,288 articles were excluded after checking for duplicates. Thus, 3,533 studies were screened of which 3,374 were excluded because they assessed different outcomes or populations, or being reviews or meta-analyses. Four papers could not be retrieved, and from the 155 studies assessed for eligibility, the final study sample included 14 papers (Figure 1).

### Methods of the selected studies

The main characteristics of the included studies are provided in Table 1. Most of them adopted a correlational study design (*n* = 11), two studies used a cross-sectional design (23, 24), and one study adopted a quasi-experimental design (25). Most studies enrolled convenience samples, with variegated levels of age and education. Data collection was performed via online platforms, in person, or in mixed online and in person modality.

Although the search was not restricted to COVID-19 vaccination attitudes, most of the studies, except for Gomes-Ng et al. (26) and Gialama et al. (27), specifically focused on vaccination hesitancy towards COVID-19. Assessment of vaccination hesitancy was performed by self-report validated scales including multiple items scored on Likert scales; five studies (28–32) adopted scales specific for COVID-19 vaccine hesitancy, namely the Multidimensional Covid-19 Vaccine Hesitancy Scale (CoVaH) (33), the Oxford COVID-19 Vaccine Hesitancy Scale (OCVHS) (34), and the COVID-19 Vaccine Acceptance Scale (VAC-COVID-19) (35).

The assessment of psychological factors associated with vaccination behaviour was most often performed via standardised self-reported scales; only two studies (25, 26) adopted specific cognitive tasks assessing decision making (Iowa Gambling Task, IGT) and cognitive flexibility (Wisconsin Card-Sorting Task, WCST). Two of the included studies closely focused on personality traits among the possible psychological underpinnings of vaccine hesitancy (12, 36), considering either the Big Five Traits (Openness to Experience, Conscientiousness, Extraversion, Agreeableness, and Negative Emotionality) (12), or the Dark Triad personality factors (Psychopathy, Machiavellianism, and Narcissism) (36). One study (23) also considered interoception and interpersonal reactiveness—as measured by the Multidimensional Assessment of Interoceptive Awareness II (MAIA) (37) and the Interpersonal Reactivity Index (IRI) (38)—in exploring differences in vaccination behaviour.

### Quality evaluation of included studies

Among the 14 studies included in the present systematic review, six were classified as unsatisfactory, seven were classified as satisfactory and one as good studies (Table 1). Major issues in quality evaluation were represented by use of convenience samples, lack of justification for sample size, partial reporting of statistical analyses, and lack of control for relevant confounders (see Supplementary Table).

### Detailed evidence description

To enhance clarity, the detailed evidence description is divided into three sections. The first section focuses on emotional dispositions, coping strategies and specific fears. The second section targets cognitive functioning, expectancies and beliefs. The third section deals with personality traits, interpersonal reactiveness and individual differences in interoceptive awareness.

### Emotional dispositions, coping strategies, and specific fears

Studies on the relationship between psychological variables and vaccine hesitancy presented a nuanced and sometimes contrastive picture depending on the population sample recruited. In this context, depressive and anxiety symptoms were the most reported factors affecting vaccine hesitancy. In Saudi Arabia, Jayakumar et al. (39) found a bidirectional association between vaccine hesitancy and mental health, indicating that higher levels of depression, anxiety, and perceived need for mental health support were both favoured by and predictive of higher vaccine hesitancy; according to the same study, in India, vaccine hesitancy was only associated with high anxiety levels. On the same line, in the Palestinian population, Veronese et al. (32) observed positive correlations between vaccine reluctance and symptoms of stress, anxiety, and depression; in this last study, depressive symptoms were a key predictor of vaccine reluctance in the West Bank and stress in East Jerusalem and among Israeli Palestinians.

However, findings from other nations presented different associations. In Japan, Harada and Watanabe (40) examined the factors associated with changes in vaccination attitudes across a five-month period (from April to September 2021) during the COVID-19 pandemic. The authors showed that some psychological variables, such as anxiety levels and risk perception, were associated with changes in vaccination attitudes, whereas higher general anxiety levels were associated with vaccine acceptance at the second time-point. Adding to this complexity, Omar et al. (30) in Pakistan reported that vaccine hesitancy was negatively associated with levels of anxiety and depressive symptoms, i.e., lower levels of symptoms were linked to higher vaccine hesitancy. In this study however, only depressive symptoms were significant predictors of vaccine acceptance. However, some null findings were also observed, as in Vicario et al. (23), where no significant predictive value for anxiety levels, alexithymia, or disgust sensitivity was found on vaccine hesitancy in an Italian sample during the post-COVID-19 period (see also Pellegrini et al.’s study for similar findings on self-report scales; Table 1). McNeil et al. (24) showed that intolerance of uncertainty was positively related with vaccine hesitancy in a group of healthy, non-anxious individuals (conversely, in a group of individuals self-reporting anxiety disturbances the intolerance of uncertainty was slightly negatively associated with vaccine hesitancy).

While the previous described studies mainly focused on anxiety and depressive symptoms, Marschalko et al. (31) explored the role of psychological flexibility, defined as the ability to accept, rather than to avoid, negative thoughts and emotions related to life circumstances, in COVID-19 vaccine uptake across different generations of women from Hungary and Romania. The findings indicated a significant and negative role of psychological flexibility in women of Generation Y (ages 26–41). Specifically, in this subsample, avoidance was a significant negative predictor of COVID-19 vaccine uptake, lowering the probability of getting vaccinated. In contrast, for Generation Z (ages 10–25) and Generation X (ages 42–64), no significant predictors related to psychological flexibility were found for COVID-19 vaccine uptake.

Beyond general affective symptoms and emotion regulation traits, specific fears (particularly those related to injections) have been identified as a contributor to vaccine hesitancy. In the UK Freeman et al. (28) observed a significant positive association between blood–injection–injury fears and COVID-19 vaccine hesitancy, envisaging a psychological barrier that should be addressed to improve vaccination programme effectiveness.

### Cognitive factors, expectancies, and beliefs

Three recent studies investigated the relationships of cognitive flexibility, decision-making under uncertainty, and dispositional optimism with vaccine hesitancy.

In a sample of New Zealand residents, Gomes-Ng et al. (26) found that lower cognitive flexibility -measured in terms of increased perseverative errors on the WCST- predicted greater personal barriers to vaccination, which encompassed moral beliefs, distrust in vaccines, and perception of one’s own health and safety or efficacy of vaccines. Findings from Gomes-Ng et al. (26) nicely fit those from Pellegrini et al. (29) in a UK sample, showing that cognitive inflexibility- higher perseverative errors on the WCST- positively predicted vaccine hesitancy.

Differences in decision-making styles and vaccination behaviour were explored in a further study by Demirci et al. (25). The authors showed that, as compared to vaccinated participants, non-vaccinated ones demonstrated worse decision-making performance, making more choices from disadvantageous and risky decks in the long run at IGT.

While the studies above focused on flexibility and decision-making as adaptive cognitive functions, Gialama et al.’s study (27) dealt with personal expectancies with regard to vaccination. In a sample of Greek community-dwelling individuals, they found that lower levels of dispositional optimism were linked to a higher lack of confidence in vaccination, reflecting concerns about vaccine trustworthiness, efficacy, and necessity.

### Personality factors, interpersonal reactiveness and interoceptive awareness

Panish et al. (12) showed that higher Openness to Experience was associated with lower vaccine hesitancy. This relationship was significantly mediated by political liberalism, suggesting that individuals higher in Openness tend to engage in pandemic mitigation behaviours, including vaccination, likely because they are more receptive to messages from politically liberal information sources promoting such efforts and discrediting conspiracy theories. Conversely, greater Extraversion consistently covaried with higher levels of vaccine hesitancy. The study reported mixed results for Conscientiousness, with a negative association with vaccine hesitancy found in only one of their two samples. Although Agreeableness correlated negatively with vaccine hesitancy in the two samples, it did not predict vaccine hesitancy in regression models. Negative Emotionality was not associated with vaccination hesitancy.

Using a similar methodology, Giancola et al. (36) explored the role of the Dark Triad on vaccine hesitancy. Although no direct association between the Dark Triad and vaccine hesitancy was observed, the authors identified a significant sequential mediated effect. Individuals with higher scores on the Dark Triad traits were more prone to believe in conspiracy theories. These conspiracy beliefs, in their turn, were negatively associated with the perception of COVID-19 risk. A lower perception of COVID-19 risk was directly related to increased vaccine hesitancy. This resulting path -from Dark Triad to conspiracy beliefs, then to reduced risk perception, and finally to increased vaccine hesitancy- suggests a complex relationship between specific personality traits and aversion to get vaccinated.

In addition to broad personality traits, individual differences in the way people tend to react to external and internal stimuli, and in their interpersonal responses, may also play a role in shaping vaccine hesitancy. Vicario et al. (23) investigated the impact of interpersonal reactiveness and individual differences in interoceptive awareness on vaccine hesitancy, and found that reduced self-reported perspective-taking and imaginative experiences contributed to vaccine reluctance. Moreover, individuals exhibiting higher vaccine hesitancy had lower scores at the not distracting scale of the MAIA questionnaire, which measures tendency not to ignore or distract oneself from sensations of pain or discomfort.

## Discussion

The present systematic review aimed at providing a deeper comprehension of vaccine hesitancy by specifically focusing on relatively stable, intrinsic psychological features in the general population. Unlike prior reviews that primarily emphasised broader sociological and cultural influences or specific, transient, vaccine- and illness-related attitudes, this review sought to identify more enduring psychological determinants, including emotional dispositions, cognitive styles and personality traits. Although the primary search was not restricted to COVID-19, almost the entirety of the studies conducted during such era explored vaccine hesitancy specifically towards COVID-19 vaccines.

The findings of this review reveal several key psychological factors potentially associated with vaccine hesitancy. In terms of emotional dispositions, the role of anxiety symptoms seems to be multifaceted: some studies indicated that anxiety and depressive levels may be associated with vaccine acceptance (30), whereas others highlighted that some specific fears, such as those related to injections (28), could explain a proportion of vaccine hesitancy cases. In terms of emotion regulation modalities, a tendency towards avoidance might negatively predict vaccine uptake in certain sub-populations. Indeed, the unique negative influence of avoidance for Gen Y females in Marschalko et al. (31) might suggest generational differences in how emotion regulation strategies affect health decisions.

As for the cognitive factors, the way individuals interpret and integrate overcoming information with their thoughts and beliefs seems to be a critical element in understanding vaccine hesitancy, particularly when individuals encounter information conflicting with their existing views. Indeed, individuals with anti-vaccination attitudes often exhibit less analytical reasoning styles and cognitive inflexibility (25–27, 29). For instance, the study from Gomes-Ng et al. (26) suggests that individuals with low cognitive flexibility may be more vulnerable to cognitive biases (e.g., confirmation bias) and less likely to engage in deliberative processing when faced with information about vaccines, in line with the findings from Pellegrini et al. (29). Moreover, the relationship between optimism and vaccine confidence reported by Gialama et al. (27) may suggest that individuals’ attitude towards life can affect their trust in health interventions and their engagement in proactive health behaviours, including vaccination. In terms of decision making processes, building on the somatic marker hypothesis (41), available findings suggest that individuals prone to vaccine hesitancy may exhibit differences in risk-taking and decision-making under uncertainty, being less guided by warning signals of potential future harm (25). Indeed, the findings by Demirci (25) suggest that non-vaccinated individuals might be more sensitive to immediate rewards than to long-term gains and less able to learning from negative feedback in uncertain situations. These findings imply that the decision to vaccinate is not always a purely rational cost–benefit analysis but can be significantly influenced by implicit processes.

In terms of the analysed personality factors, evidence suggests that some specific malevolent personality traits may predispose individuals to embrace misleading narratives, which in turn diminish their perceived threat from diseases and foster vaccine reluctance (36). Conversely, intellectual humility, characterised by openness to re-evaluate one’s beliefs, has been linked to more favourable vaccine attitudes and intentions. Moreover, other traits like lower agreeableness and lower dispositional optimism have been associated with a lack of confidence in vaccination (12). The finding that greater extraversion consistently covaried with higher levels of vaccine hesitancy (12), also aligns with theories linking this trait to risky behaviours (42, 43). Overall, these studies support the notion that personality influences vaccine hesitancy, underscoring the importance of considering different personality taxonomies and their specific mediating psychological mechanisms when addressing vaccine hesitancy.

The findings from the present review nicely complement, and extend, the available body of evidence on vaccine hesitancy gathered from previous syntheses of the literature, thus providing relevant theoretical implications. Previous reviews, such as those by Romate et al. (10), Pourrazavi et al. (13), Rizzo et al. (4), and Blukacz et al. (5), largely focused on immediate, context-specific beliefs (e.g., vaccine safety and efficacy, trust in specific institutions, or concerns about rapid vaccine development) and broader sociological or cultural factors. These factors are undeniably important, but previous reviews did not delve into the underlying stable psychological predispositions that shape how individuals process information, perceive risks, and form their vaccine attitudes. By specifically focusing on enduring emotional dispositions, personality traits, and cognitive features, the present paper identified some fundamental individual differences contributing to understand the reasons why these factors affect vaccination hesitancy at the individual level. For instance, mistrust in authority specialists (5, 10) might be more pronounced in individuals with specific personality profiles, like those scoring high on Dark Triad traits. Similarly, the role of perceived risk was identified in other reviews (5, 10), but the present findings might help further exploring its cognitive roots, linking it to the impact of conspiracy beliefs and distinct reasoning styles (27, 36). By focusing on these stable, intrinsic psychological characteristics, this review contributes to a more nuanced and psychologically based comprehension of vaccine hesitancy, providing clues on individuals *who* might be more prone to hesitancy due to their psychological profile, rather than just because of *what* beliefs they hold.

As described above the studies included in the review have some limitations. A major drawback was related to the recruitment strategy adopted by the included studies, which mainly relied on convenience samples and online survey administration. This might have limited the observation to specific users, providing a partial view on the targeted phenomenon. Similarly, the inherent risk of social desirability related with the use of self-report measures in many primary studies might have potentially affected the accuracy of reported psychological states and attitudes. Furthermore, most original studies employed correlational or cross-sectional designs, which limited the possibility to establish causal relationships between the identified psychological factors and vaccine hesitancy. The use of longitudinal designs in future investigations might allow to ascertain how stable psychological factors modulate vaccination behaviours. While the heterogeneity of the nationality samples considered could represent an advantage of the gathered findings, this could also be responsible for some inconsistent results among them. Future research might explore more closely the cross-cultural factors associated with the vaccination behaviour.

A couple of limitations are inherent to the present systematic review itself. The first is related with the eligibility criteria adopted, which allowed to gather from the literature some transversal, broadly general, psychological factors associated with vaccine hesitancy but explicitly excluded some specific populations and conditions of possible interest in the scientific research on vaccination behaviour (e.g., parents, caregivers, healthcare professional and specific clinical populations). Thus, future syntheses of the literature are warranted to provide information on such specific populations. Second, because of the time frame for literature search adopted here, most studies retrieved in the present work addressed vaccine hesitancy for COVID-19, but it would be of interest to compare psychological factors affecting vaccination intention with reference to other medical conditions. Third, although this review targeted key psychological factors related to vaccine hesitancy, future research should address additional influences such as mistrust, misinformation, and social dynamics that may even indirectly shape vaccination attitudes and behaviours (10, 13).

The evidence gleaned from this review provides important implications for interventions aimed at increasing vaccine acceptance. On the basis of the identified role of the affective, cognitive and personality factors described above, the interventions should move beyond generic information campaigns and adopt more tailored, personalised communication strategies (44, 45). These interventions might emphasise collective responsibility and the social benefits of vaccination in individuals with high levels of avoidance or low levels of cognitive empathy. Cultivating more analytical processing and fostering intellectual humility might be a target of interventions for individuals prone to intuitive reasoning or those who present dark personality traits. Ultimately, a transdisciplinary approach that integrates psychological understanding with public health strategies will be essential for developing effective interventions that not only address immediate concerns but also consider the deeply rooted, stable psychological factors influencing vaccine hesitancy globally.

## Data Availability

No original dataset was generated in this review; all data extracted from the included studies are reported in the article and its Supplementary material. Further inquiries can be directed to the corresponding author.

## Footnotes

^1^ https://www.prisma-statement.org/
