# Supplementary material for: A systematic review on the psychological factors behind vaccine hesitancy in the COVID-19 era
Source: Front Public Health. 2025 Nov 11;13:1711428. doi: 10.3389/fpubh.2025.1711428 (PMC12644076; doi:10.3389/fpubh.2025.1711428)
Supplement: Supplementary file 1 [file Table_1.DOCX]

**Newcastle-Ottawa Scale (NOS; Wells et al., 2000) adapted for the present systematic review**

**Selection - Maximum 6 stars**

*Representativeness of the sample*:

a) Truly representative of the target population *

b) Somewhat representative of the target population *

c) Selected group of users or convenience sample (e.g.: nurses, volunteers)

d) No description of the sampling strategy

*Sample size:*

a) Justified and satisfactory *

b) Satisfactory (at least 100 participants for each measure) *

c) Not justified or satisfactory or no information provided

*Selection of the controls*:

a) Between-subject studies: matched sample for age, sex, education *

b) Correlational studies or not matched in between-subject studies

*Measurement of psychological variables:*

a) Validated measurement tool **

b) Non-validated measurement tool, but the tool is available or described *

c) No description of the measurement tool

**Comparability - Maximum 1 star**

*Confounding factors controlled:*

a) Data/ results adjusted for relevant predictors/risk factors/confounders (education, age, socioeconomic status, medical conditions) *

b) Data/results not adjusted for all relevant confounders/risk factors/information not provided

**Outcome - Maximum 3 stars**

*Assessment of the outcome:*

a) Double blind assessment **

b) Single blind *

c) No description

*Statistical test:*

a) The statistical test used to analyze the data is clearly described and appropriate, and the measurement of the association (including confidence intervals or: effect size, non-significant effects; all value of statistic methods; mean and SD or ES) are presented (if correlation, effect size not required) **

b) Tests are appropriate, there is p value all value of statistic methods, but reporting is incomplete *

c) The statistical test is not appropriate or not described

**TOTAL**

a) Very Good Studies: 9-10 stars

b) Good Studies: 7-8 stars

c) Satisfactory Studies: 5-6 stars

d) Unsatisfactory Studies: 0 to 4 stars

**Supplementary Table**. Summary assessment of including studies based on the NOS (studies in alphabetical order)

| **Study** | **Selection** | | | | **Comparability** | **Outcome** | | **Total** |
| --- | --- | --- | --- | --- | --- | --- | --- | --- |
|  | *Representativeness* | *Sample size* | *Controls* | *Variables* | *Confounding* | *Blinding* | *Statistics* |  |
| Demirci et al., 2023 | - | - | * | ** | - | - | * | 4 * |
| Freeman et al., 2023 | * | * | - | ** | - | * | ** | 7 * |
| Gialama et al., 2024 | - | * | - | ** | - | - | ** | 5 * |
| Giancola et al., 2024 | - | * | - | ** | - | - | ** | 5 * |
| Gomes-Ng et al., 2024 | - | * | - | * | - | - | ** | 4 * |
| Harada et al., 2022 | - | * | - | ** | - | - | ** | 5 * |
| Jayakumar et al., 2022 | - | * | - | ** | - | - | ** | 5 * |
| Marschalko et al., 2022 | - | * | - | ** | - | - | ** | 5 * |
| McNeil et al., 2022 | - | - | - | ** | - | - | ** | 4 * |
| Omar et al., 2024 | - | - | - | ** | - | - | ** | 4 * |
| Panish et al., 2023 | - | * | - | ** | - | - | ** | 5 * |
| Pellegrini et al., 2024 | - | - | - | ** | - | - | ** | 4 * |
| Veronese et al., 2023 | - | * | - | ** | - | - | ** | 5 * |
| Vicario et al., 2024 | - | - | * | ** | - | - | * | 4 * |

*Note*. According to the NOS scoring system: Very Good Studies: 9-10 *, Good Studies: 7-8 *, Satisfactory Studies: 5-6 *, Unsatisfactory Studies: 0-4 *
